# Isolated pulmonary valve infective endocarditis in isolated congenital supravalvular pulmonary stenosis: a case report

**DOI:** 10.1186/s12872-025-05267-6

**Published:** 2025-12-01

**Authors:** Nicholas E. Kunce, Adam J. Kisling, Lisa M. Conte, Nitin Rao, Travis E. Harrell

**Affiliations:** 1 Department of Medicine, Walter Reed National Military Medical Center, Bethesda, MD USA; 2 Department of Medicine, Division of Cardiology, Walter Reed National Military Medical Center, Bethesda, MD USA

**Keywords:** Case report, Infective endocarditis, Pulmonary valve infective endocarditis, Supravalvular pulmonary stenosis, Pulmonary valve stenosis, Adult congenital heart disease

## Abstract

**Background:**

Isolated pulmonary valve stenosis, of which supravalvular pulmonary stenosis is a subcategory, rarely occurs in live births. Isolated pulmonary valve infective endocarditis is uncommon and typically arises in settings of recurrent transient bacteremia, immunodeficiency, or congenital heart disease. Any infective endocarditis carries high morbidity with complications commonly including congestive heart failure, acute renal failure, and septic emboli among others. Consistent with previously published cases of pulmonary valve infective endocarditis, these cases can be challenging to diagnose and treat.

**Case presentation:**

We present the case of a 52-year-old male with well-controlled human immunodeficiency virus on antiviral therapy and known non-syndromic congenital supravalvular pulmonary stenosis who developed pulmonary valve infective endocarditis. He originally presented to the emergency room with acute delirium and was diagnosed with *Streptococcus agalactiae* meningitis and bacteremia due to suspected pharyngitis, but despite antibiotics, progressed to meningoencephalitis and septic shock. His transthoracic echocardiogram was without evidence of infective endocarditis; however, a transesophageal echocardiogram was performed due to a high index of suspicion and confirmed the diagnosis. The patient subsequently required right ventricular outflow tract pericardial patch repair and replacement of the pulmonary valve with a bioprosthetic valve. At follow-up, he was asymptomatic and tolerating rehabilitation.

**Conclusions:**

Pulmonary valve infective endocarditis can be difficult to diagnose because of limited sensitivity on transthoracic echocardiography and often underappreciated predisposing factors such as simple congenital heart disease. This case provides a reminder to providers of a rare, life-threatening entity that requires a high index of suspicion to diagnose.

## Background

Isolated pulmonary valve (PV) stenosis is typically a congenital disease that occurs in roughly 2 per 2000 live births worldwide, accounting for 7–12% of congenital heart diseases [1]. PV stenosis can be characterized as valvular, subvalvular, or supravalvular. In supravalvular pulmonary stenosis (SVPS), the functional obstruction lies in the pulmonary artery which can arise from the main artery, bifurcation point, distal branches, or a combination of the three [2]. SVPS can encompass either a focal constriction within one or more arteries or a broader underdevelopment of the distal pulmonary arterial network [3]. Although patients with PV stenosis are generally asymptomatic, they can develop fatigue and dyspnea. In the most severe form of PV stenosis, angina can develop but usually in the setting of conditions that cause hemodynamic instability [4]. One potential complication of PV stenosis, along with other congenital heart diseases, is infective endocarditis (IE). Involvement of the PV is rare, occurring in 1.5–2.5% of all cases of IE, and it is even more uncommon in the absence of involvement of another valve [5]. It typically occurs in settings of recurrent transient bacteremia, intravenous drug use, immunodeficiency, presence of intracardiac devices, or an abnormal PV [6–8]. Complications of any IE can be devastating and include local cardiac complications, embolic phenomena, metastatic spread, and acute renal failure. In this report, we describe a case of isolated PV IE in the setting of isolated congenital SVPS.

## Case presentation

We present a case of a 52-year-old male with known non-syndromic congenital SVPS and well-controlled human immunodeficiency virus (HIV) on highly active antiretroviral therapy (HAART) with daily bictegravir/emtricitabine/tenofovir alafenamide. His SVPS had been diagnosed in childhood and routinely monitored throughout his adult life with transthoracic echocardiograms (TTE) while remaining asymptomatic. Ten years before presentation, computed tomography (CT) of his chest demonstrated a dilated main pulmonary artery (PA) and a dilated left PA. Four years before his presentation, a TTE and subsequent cardiac magnetic resonance imaging (CMR) showed mild right ventricular hypertrophy, mild SVPS, mild PV regurgitation, and a dilated main pulmonary artery. The patient was also routinely followed by Infectious Disease consultants while on HAART. Soon before his presentation, he had an undetectable HIV viral load and CD4 count of 620 cell(s)/mcL (normal: 491–1734). Furthermore, he had no documented substance abuse history and consistently tested negative for illicit substances per routine workplace screening.

On the index presentation, he was found incoherent at home and brought to the emergency department, where he experienced a generalized tonic-clonic seizure and required intubation. Laboratory testing revealed leukocytosis and acute renal failure. Two sets of blood cultures were drawn and were positive for *Streptococcus agalactiae*. An unenhanced CT of the patient’s brain on admission was without any acute intracranial findings. Given the high concern for meningitis, a lumbar puncture was performed, and both polymerase chain assay and culture were positive for *S. agalactiae*. The patient was admitted to the intensive care unit and treated for meningitis with intravenous ceftriaxone, with a clinical course complicated by acute renal failure managed with continuous veno-venous hemodialysis, subsegmental pulmonary embolism, and seizures. His HIV viral load on admission was undetectable. A TTE obtained shortly after admission was comparable to his TTE from four years prior including stable mild PV regurgitation, but it did not clearly visualize the PV structure. A whole-body positron emission tomography (PET) CT was also later performed to evaluate for a nidus for infection but demonstrated no distinguishable infectious source. Unenhanced magnetic resonance imaging of the brain demonstrated evidence of bacterial meningoencephalitis to include ventriculitis, purulence on the frontal sulci, cysts/adhesions in the subarachnoid space, and a single microhemorrhage in the left frontal subcortical white matter. Overall, his *S. agalactiae* meningoencephalitis and bacteremia were thought to be a sequela of bacterial pharyngitis.

**Fig. 1:**
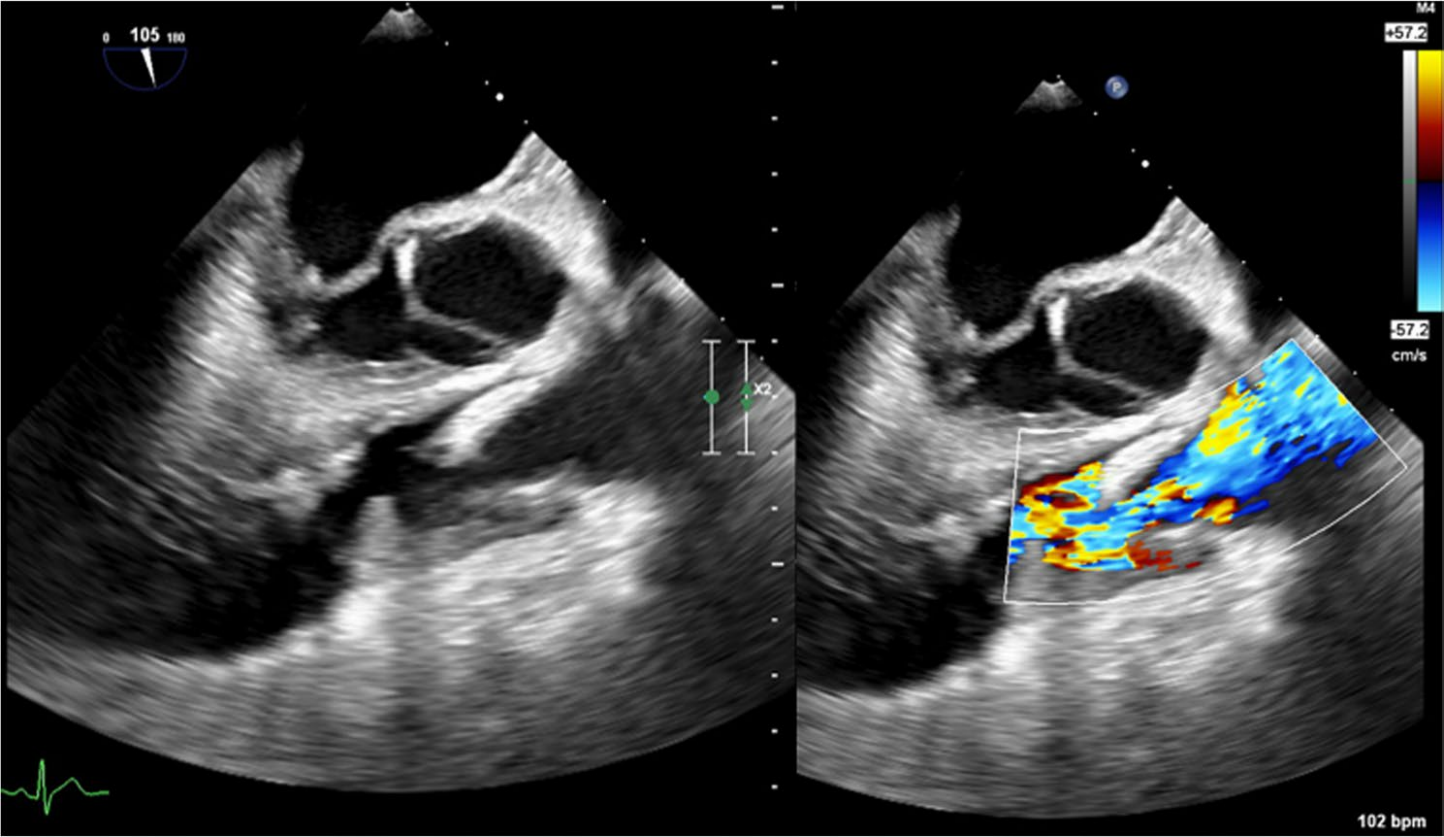
TEE in a mid-esophageal view with simultaneous 2D and color flow doppler demonstrating PV thickening, a 21 mm x 7 mm vegetation, and severe PV regurgitation

The patient clinically improved and was subsequently extubated, however, despite continued treatment with intravenous ceftriaxone, he developed septic shock. Evaluation for IE was pursued given his history of isolated SVPS. Cardiac auscultation at the time revealed his baseline III/VI systolic murmur loudest at the left upper sternal border. Additionally, there were no cutaneous manifestations of IE, and a CT of his chest showed no septic emboli. A TTE was repeated and demonstrated mild PV regurgitation and moderate SVPS without evidence of IE. Due to a high index of suspicion, a transesophageal echocardiogram (TEE) was performed, demonstrating a thickened PV, along with a 21 mm x 7 mm vegetation, and severe PV regurgitation (Fig. 1) which confirmed the diagnosis of PV IE. Multiple sets of blood cultures drawn at that time and thereafter yielded no growth.

**Fig. 2:**
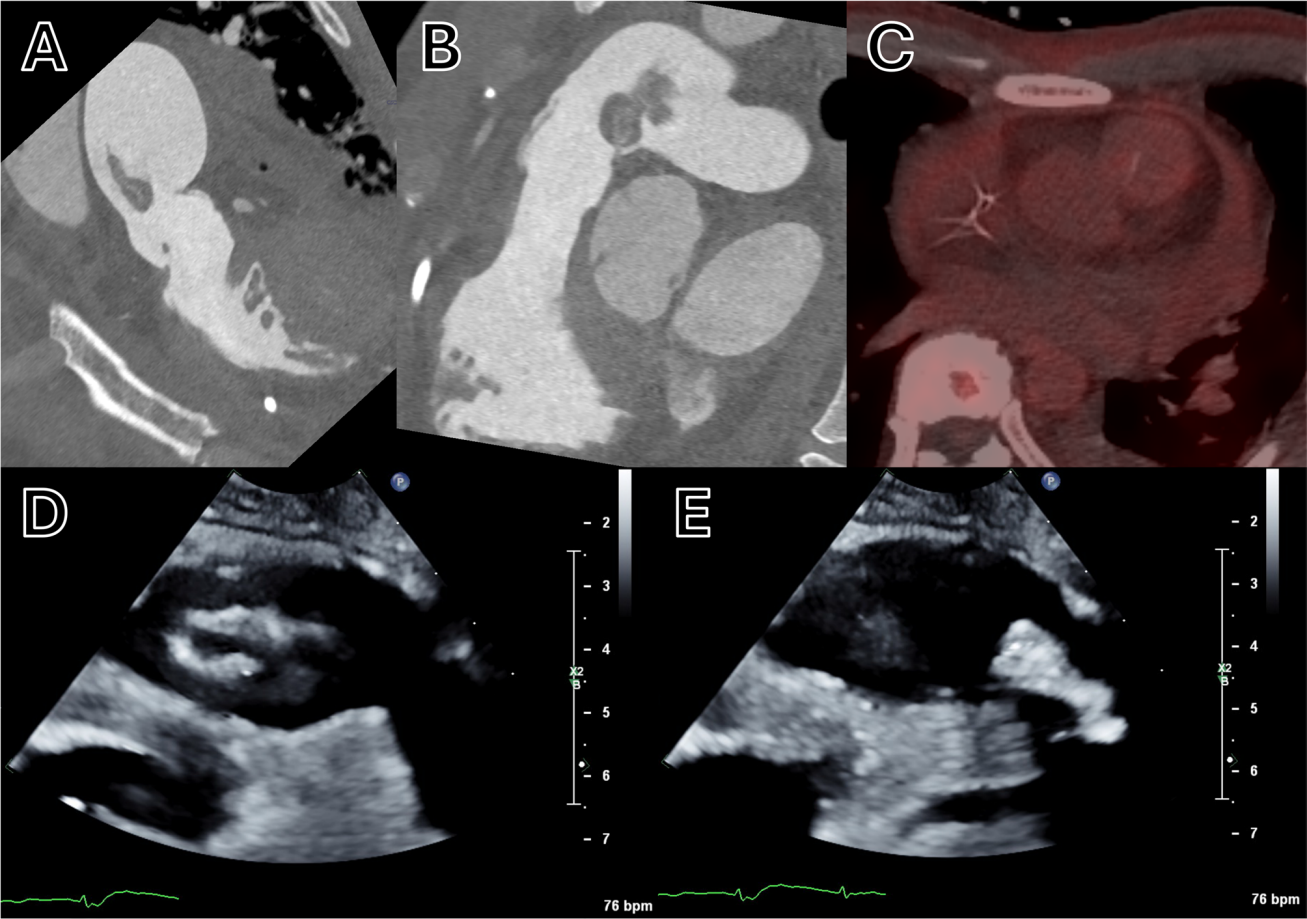
**A** + **B** CT angiography with two perpendicular cross-sectional reconstructions of the RVOT demonstrating destruction of the PV and a large mobile vegetation. **C** PET CT without hypermetabolic activity on the PV. **D** + **E** TTE in parasternal short-axis of the RVOT demonstrating vegetation motion in diastole and systole

**Fig. 3:**
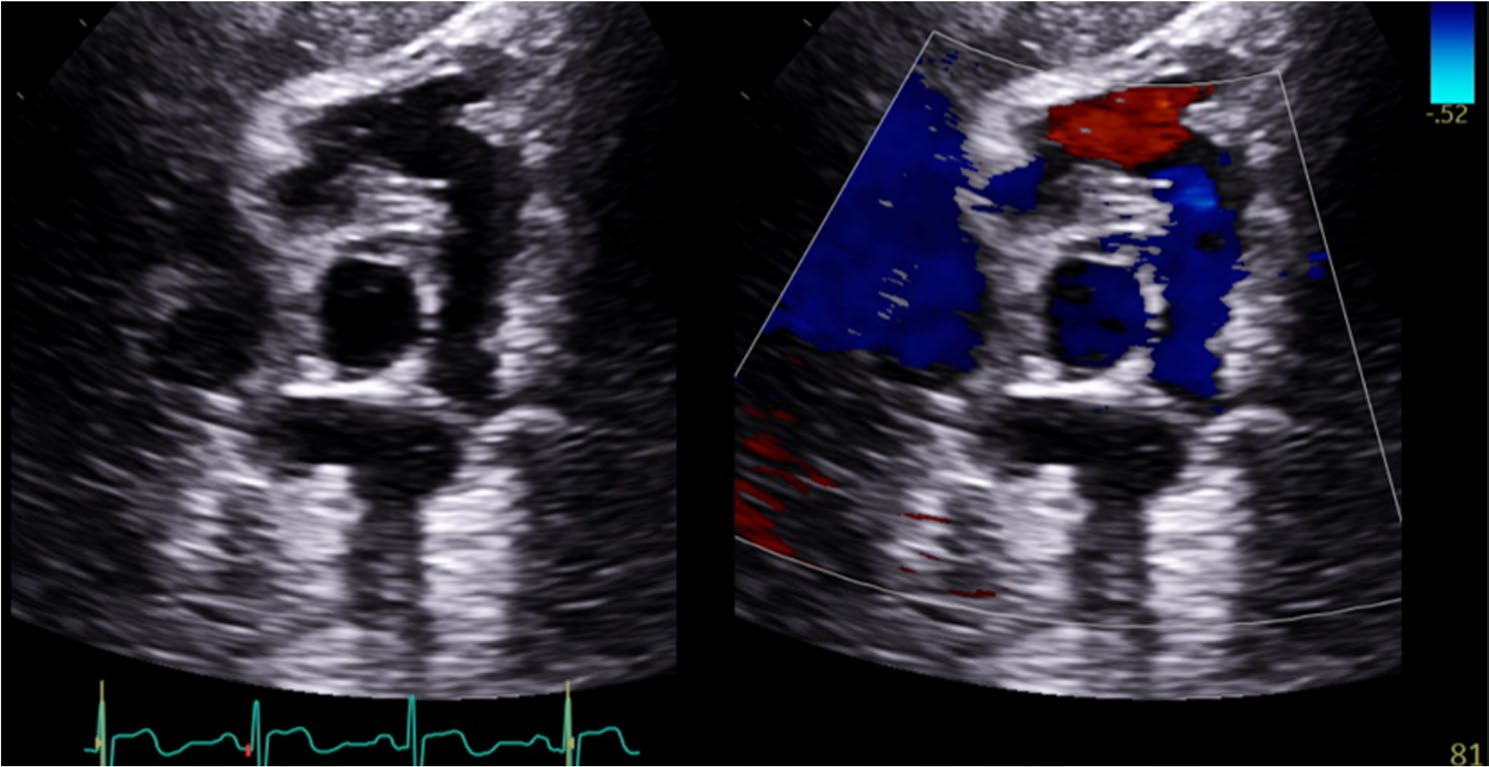
Post-operative TTE in modified parasternal short-axis with simultaneous 2D and color doppler of the PV demonstrating PV/RVOT repair. No PV regurgitation or stenosis was demonstrated in this study

A multidisciplinary heart team meeting was held and determined that PV replacement was the optimal therapeutic option given the size of the vegetation and the severity of regurgitation. Surgery was deemed to be nonurgent with a plan for continued treatment with intravenous ceftriaxone in the interim. Prior to surgery, the patient underwent CT angiography of the lungs and coronary arteries which demonstrated destruction of the PV and a large mobile vegetation (Fig. 2, Panels A + B), a repeat PET CT scan which was without hypermetabolic activity on the PV (Fig. 2, Panel C), and a focused TTE of the right ventricular outflow tract (RVOT) which demonstrated a mobile vegetation (Fig. 2, Panels D + E). He subsequently underwent a PV/RVOT pericardial patch repair with implantation of a 29 mm Edwards Magna Ease valve, as demonstrated on TTE (Fig. 3) performed 4-weeks post-operatively. PV aerobic and anaerobic cultures at the time of surgery were without growth. At his 4 and 8-week post-operative follow-up appointments, he was cardiovascularly asymptomatic and tolerating rehabilitation.

### Discussion and conclusions

We present the case of a middle-aged male with known isolated SVPS and well-controlled HIV on HAART who was found to have *S. agalactiae* meningoencephalitis and isolated bacterial IE of the PV that were thought to be secondary to untreated bacterial pharyngitis. His course was complicated by acute renal failure, pulmonary embolism, and meningoencephalopathy with seizures. TEE proved to be critical in the diagnosis of IE by visualizing the vegetation on the PV. Despite an extensive and lengthy hospital course, the combination of medical and surgical management led to a favorable outcome.

As previously described, PV IE is not common and data is limited to case reports and small case series. As such, making a correlation between our patient’s isolated SPVS and his PV IE was difficult. Congenital heart disease is a risk factor for IE, and inferences may be made by regarding SVPS as a form of congenital heart disease and it therefore leading to an increased risk of native valve endocarditis. There is an estimated risk of IE in native PV stenosis of 0.2% per 1000 patient-years [9]. SVPS is a rare entity that can be identified in isolation or with other congenital heart diseases such as Williams Syndrome or Tetralogy of Fallot. In some instances, SVPS can be acquired after surgical intervention involving the main pulmonary artery [2, 10]. As seen with our patient, a common characteristic of SVPS is a stenosing ridge of tissue just above the sinotubular junction of the main pulmonary artery. This aberrant structure may cause turbulent blood flow and result in continual trauma to and eventual thickening of the PV [10]. We hypothesize that this turbulent flow coupled with subsequent maladaptive remodeling served as a nidus for IE.

Bacterial IE is well known in the literature to carry high rates of morbidity and mortality. Since clinical manifestations can vary from acute to subacute, making the diagnosis early to prevent complications can be difficult. Echocardiography remains one of the initial tools for diagnosis, however, surface echocardiography of the PV can be difficult depending on the patient’s habitus and the sonographer’s skill. As was seen in the case of our patient, TTE was insufficient to diagnose PV IE both at the time of presentation and when clinical suspicion for IE was high. TEE provided superior visualization of this anterior and right-sided structure and led to our final diagnosis. The use of TEE in isolated PV IE cases has been cited multiple times [5, 8], and this case further supports its utility when TTE is non-diagnostic or clinical suspicion for IE remains high.

*S. agalactiae*, also known as group B streptococcus, colonizes the throat, rectum, and female genital tract. Among cases causing bacteremia, around 2–9% will present as endocarditis which is characterized as an acute onset, presence of large vegetations, rapid valvular destruction, and frequent complications [11]. Isolated PV IE caused by *S. agalactiae* diagnosed by TEE has been reported previously and successfully treated with intravenous antibiotics [12]. In the case of our patient, we presume he acquired this infection via the oropharyngeal tract leading to seeding of the bloodstream. We further presume that the patient’s HIV status did not play a role in his susceptibility to acquiring this infection as he was adherent to HAART and immunocompetent with an undetectable viral load at the time of presentation. Surgical valve replacement in addition to intravenous antibiotics was determined as the clear avenue for treatment based on the large size of the vegetation, underlying congenital heart disease, and the valve involved.

This unusual case of isolated PV IE in a patient with isolated SPVS demonstrates the severe morbidity of IE and the challenges presented in making the diagnosis. Recognizing signs and symptoms of IE and keeping a high index of suspicion for IE when the patient’s clinical picture is ambiguous, should warrant prompt evaluation with tools such as echocardiography to appropriately diagnose. Furthermore, utilizing TEE when TTE is non-diagnostic for PV IE, remains useful in visualizing vegetations and assessing the valvular sequelae of PV IE.

## Data Availability

No datasets were generated or analysed during the current study.

## Abbreviations

PV: Pulmonary valve
SVPS: Supravalvular pulmonary stenosis
IE: Infective endocarditis
HIV: Human immunodeficiency virus
HAART: Highly active antiretroviral therapy
TTE: Transthoracic echocardiogram
CT: Computed tomography
PA: Pulmonary artery
CMR: Cardiac magnetic resonance imaging
PET: Positron emission tomography
TEE: Transesophageal echocardiogram
RVOT: Right ventricular outflow tract

## Declarations

### Duplicate publications

The authors declare that the content of the manuscript has not been published or submitted for publication elsewhere. The authors declare that abstracts with consolidated content related to this case were presented at the following conferences: the Mid-Atlantic Capital Cardiology Symposium (November 2023) and the American College of Cardiology Annual Scientific Session (April 2024).

### Disclaimer

 The opinions and assertions expressed herein are those of the author(s) and do not reflect the official policy or position of the U.S. Government, Department of Defense, Defense Health Agency, Uniformed Services University of the Health Sciences, Department of the Army, or Department of the Navy.
